# Microfluidic paper–based analytical extraction devices (µPAEDs): a cost-effective and portable solution for biomarkers, contaminants and VOC detection

**DOI:** 10.1007/s00604-025-07333-4

**Published:** 2025-07-05

**Authors:** Shruti Janakiraman, Reshmi Saravana Bhava, Naresh Kumar Mani

**Affiliations:** 1 Microfluidics, Sensors and Diagnostics (μSenD) Laboratory, Centre for Microfluidics, Biomarkers, Photoceutics and Sensors (µBioPS), Department of Biotechnology, Manipal Institute of Technology, Manipal Academy of Higher Education, Manipal, Karnataka 576104 India; 2 Innotech Manipal, Manipal Institute of Technology, Manipal Academy of Higher Education, Manipal, Karnataka 576104 India

**Keywords:** Extraction, Biomarkers, Contaminant determination, Microfluidics, VOCs, µPADs, µPAEDs

## Abstract

The human body relies on various biological and environmental cues, and regulatory processes for basic functioning and maintenance of health. Thus, by monitoring these cues, which include biomarkers and chemical contaminants, scientists and health professionals can prioritize the health, well-being, and longevity of the general population. These analytes are found in biological and other fluids and are usually extracted using conventional extraction methods like liquid–liquid or solid-phase extraction. Until recently, these methods were used in tandem with conventional detection techniques like LC–MS/MS and GC–MS/MS. However, this inept combination proves to be disadvantageous for the general population due to its higher costs, requirement of specialized equipment and experienced personnel, usage of several organic and environmentally harmful reagents, and a centralized approach. Thus, recently there has been a rise in the usage of microfluidics, particularly µPADs and conventional extraction techniques. By capitalizing on the multifarious advantages of µPADs, including affordability, miniaturization, and dispensable equipment, researchers have developed all-in-one microfluidic paper–based analytical extraction devices (µPAEDs). This approach mitigates the risks associated with the former and improves costs, transportability, and accessibility. Additionally, this novel approach has also been applied to non-invasive detection of volatile organic compounds (VOCs). In this review, we highlight the advancements of µPAEDs to detect myriads of biomarkers and chemical contaminants in samples like blood, breath, and water.

**Graphical Abstract:**

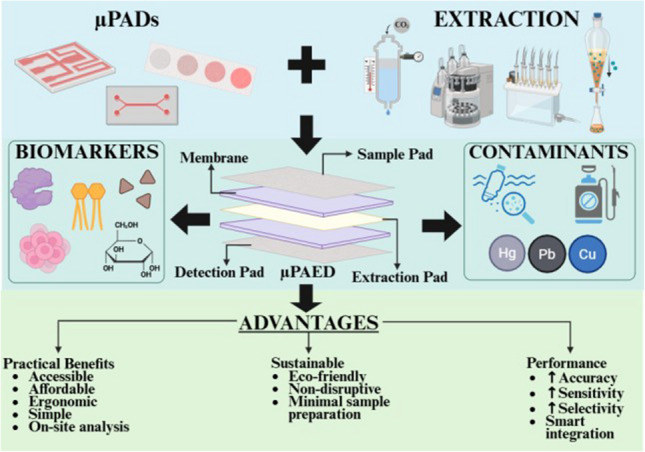

## Introduction

Biological markers, also called biomarkers, are often roughly defined as objective biological cues indicative of the subject’s health [1–5]. It is also measured as a response to therapeutics or environmental exposure to certain elements [1, 3, 4]. Despite the unchanging nuances, a cursory search of literature highlights their dynamically changing definitions. The U.S. Food and Drugs Administration (FDA) and National Institute of Health (NIH) jointly launched the Biomarkers, EndpointS and other Tools (BEST) glossary to combat this glaring disparity. With the help of this tool, they defined seven biomarker categories: susceptibility, monitoring, diagnostic, prognostic, predictive, response, and safety [1, 3]. Additionally, biomarkers have also been classified based on their type into proteins, small molecules, carbohydrates, lipids, hormones, and nucleic acids. To apply these biomarkers in clinical settings, they must first be subjected to a rigorous qualification process. A clinically approved biomarker is consistent in its association with the disease, regardless of its presentation. It should also provide new information which can be integrated with existing diagnostic tests, thus improving their clinical efficiency. And most importantly, it should enhance the healthcare professional’s managing capacity and efficiency [1, 4]. Although these conditions may sound simple to attain, the qualification process is rigorous as most biomarker candidates fail to fulfill them. Nonetheless, biomarkers have been applied in various sectors, with diagnostic and health monitoring sectors seeing a recent rise in popularity. In these sectors, levels of singular or various biomarkers are measured and monitored in a continuous and real-time capacity in both healthy and diseased populations. Some conditions which can be monitored in this manner include acute cardiac injury, heart failure, chronic obstructive pulmonary disease (COPD), idiopathic pulmonary fibrosis, inflammatory bowel disease, diabetes, systemic lupus, autoimmune diseases, and cancer [4, 5].

In addition to biomarkers, chemicals like pesticides and plasticizers have also been detected in body fluids like blood, plasma, sweat, saliva, and urine [6]. Their occurrence in these samples can be traced to ingestion, inhalation, or dermal exposure to contaminated food, drinking water, and environmental and occupational exposure [7]. These chemicals, also known as chemical contaminants, cover a wide range of chemicals from naturally occurring elements and mycotoxins to pesticides and chemicals in effluents and run-offs [8, 9]. Chemical contaminants commonly found include pesticides and plasticizers. A broad spectrum of diseases has been associated with exposure to these contaminants including endocrine disruption, neurological disorders and diseases, fetal defects, disruption of immune, metabolic, memory storage and nerve excitability functions, dementia, various organ damage, Wilson’s disease, various cancers, along with vomiting, nausea, vertigo, tremors, pneumonia, asthma, and reproductive issues [10–14]. Additionally, some of these contaminants have been classified as carcinogenic and probably carcinogenic substances. Thus, it is extremely essential that health professionals detect these chemicals as early as possible to prevent fatal outcomes.

Both biomarkers and chemical contaminants have been found in various samples like blood, sweat, saliva, water, and foodstuff. Samples collected from these sources should be pre-treated prior to detection. This is usually performed with various extraction techniques such as liquid–liquid extraction (LLE); solid-phase extraction (SPE); Quick, Easy, Cheap, Effective, Rugged, and Safe (QuEChERS); dispersive SPE (d-SPE); and leaching. After extraction, samples are detected through conventional methods like LC–MS/MS, GC–MS/MS, and HPLC–DAD, among others. Until recently, this was the approach taken for detecting analytes in samples. However, this combination is detrimental for a wide population due to its high cost, requirement of skilled professionals for handling and operation, and laborious nature. Thus, such testing is performed only in centralized testing facilities. Furthermore, it is not portable and thus cannot be accessed nor operated in resource-limited and remote areas. Particularly, liquid–liquid extraction (LLE) is the most common type of extraction performed for detection through conventional channels. But it requires large volumes of reagents and toxic chemicals.

**Fig. 1:**
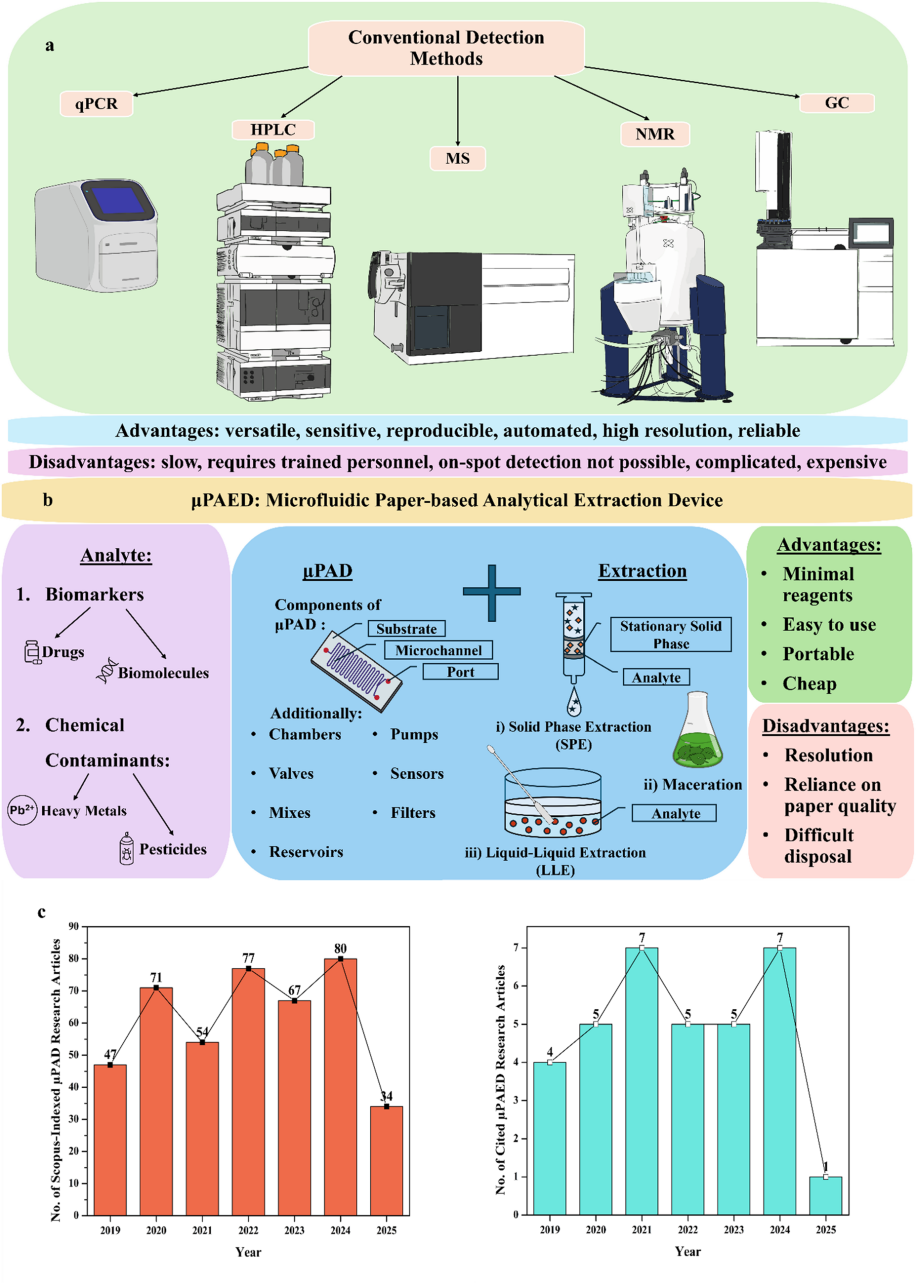
**a** The conventional methods considered to be the gold standard for the detection of various analytes (not depicted here) have been listed alongside their advantages and disadvantages. **b** µPAEDs integrating the various components of µPADs and extraction processes have been depicted. Left: Commonly detected analytes (biomarkers and chemical contaminants) have been listed. Right: The advantages and disadvantages of this approach have been described. **c** Left: The number of research articles from Scopus-indexed journals relating to µPADs, published between 2019 and 2025, has been depicted as a bar graph. This data was obtained by entering the keywords: “μPAD,” “microfluidic paper-based device,” and “microfluidic paper based device.” Right: The number of relevant research articles pertaining to µPAEDs that have been published between 2019 and 2025 and cited in this work has been graphically represented

In microfluidics, designs or patterns are fabricated/printed/embossed onto a substrate and fluid flow is manipulated through them, enforcing complete mixing and reaction at microlevel. In this approach, accuracy, precision, and sensitivity are enhanced [15–17]. Although there are various branches in this field with each boasting significant advancements, for the purpose of this review, paper-based microfluidics is considered. Taking advantage of the several benefits of using paper including its lightweight, eco-friendly, cost-effective and absorbent features, researchers developed microfluidic paper–based analytical devices (µPADs) by leveraging paper [18]. Due to their chemical excellence, self-driven, user-friendly, simplistic design, and rapid and accurate analysis, µPADs have been applied for the detection of various analytes such as air pollutants, pesticides, antibiotics, plasticizers, nucleic acids, carbohydrates, antibodies, proteins, and hormones [15, 16, 19–27]. In recognition of these advantages, researchers shifted their attention towards the integration of conventional extraction techniques with µPADs. This combination results in an all-in-one device which is affordable, portable, and accessible, called the microfluidic paper–based analytical extraction device (µPAEDs). In this review, the recent innovation and developments in this field have been discussed (Fig. 1). As this field is recently developed, its several disadvantages, such as the resolution of the device and proper disposal of solvents, are challenges that are yet to be overcome. By utilizing µPAEDs, both the volume of solvents used and the extraction time are minimized because of the miniaturization and optimization of the extraction process. Furthermore, due to the integration of extraction with the detection device, the time lapsed between the two processes is reduced significantly. Additionally, these µPAEDs have also been applied for non-invasive detection of volatile organic compounds (VOCs) for facilitating a truly decentralized approach that has been highlighted as well.

### Detection of analytes with µPAEDs

While there are several methods for the separation of the molecule of interest from the bulk matrix, extraction is one of the most used techniques for complex mixtures. Extraction can be simply defined as the transfer and concentration of the analyte from one phase to another immiscible phase based on differential solubility across the phase boundaries [28]. Liquid solvents are usually used as samples, while other solvents or solids are used for the extraction phase. Conventional extraction methods, such as maceration, percolation and decoction, are simple and cost-effective [29]. Specialized extraction methods like microextraction yield a high concentration of the analyte while also reducing the amount of extraction phase used [28]. To date, different extraction methods have been employed in conjunction with various detection techniques. This approach has been applied to paper-based microfluidics, as detailed in the previous section. Despite its several advantages, this approach is unsuitable for field testing as extraction steps can only be performed in centralized laboratories. Moreover, it is expensive and time-consuming and requires complex and specialized equipments to operate. Until recently, microfluidics and especially microfluidic paper–based analytical devices (µPADs) were used mostly for detection purposes [30–33]. After extraction, the extracted solvent was deposited onto the µPAD which carried out the detection through electrochemical, optical or other means. Still, this approach could not overcome the necessity of centralized laboratories for its completion. Naturally, several researchers attempted and developed all-in-one µPADs. These all-in-one devices integrate extraction and detection systems into a single entity. In this review, we refer to these devices as microfluidic paper–based analytical extraction devices (µPAEDs). In fact, on-site extraction and detection can be manifested through µPAEDs, minimizing sample preparation steps significantly. Their portable and miniaturized design also boasts specificity, sensitivity and selectivity comparable to conventional approaches, thus permitting wide public access and acceptance. The usage of paper in their design effectively decreases the overall costs, reducing economic burdens on users. Furthermore, these devices adopt a decentralized approach due to integration of extraction and can be on-the-spot and point-of-care (POC) friendly. To the best of our knowledge, this is the first review which discusses the latest developments in this area at length. In this section, the recent innovations in the field for the detection of biomarkers and chemical contaminants in various sample types has been discussed (Table 1).

**Table 1:** List of analytes detected by µPAEDs along with extraction, printing, cutting, and detection methods

Sr. no	Analyte	Extraction method	Sample used	Type of paper used	Printing method	Cutting method	Detection method	LOD	Ref
1	Ascorbic acid	Solid-phase extraction (SPE)	Whole human blood	Whatman No. 1 filter paper and LF1 membrane	Wax dipping	NA	Electrochemical	2.44 µM	[34]
2	Cancer biomarker: carcinoembryonic antigen (CEA)	MIP-facilitated SPE	Human serum	Whatman No.1 filter paper	Wax printing	Manual cutting	Electrochemical	0.32 ng/mL	[35]
3	3-nitrotyrosine (3-NT) and 4-nitroquinolin-N-oxide (4-NQO)	MIP-facilitated SPE	Human serum, human urine, whole blood	Whatman No.1 filter paper	Wax printing	Possibly manual cutting	Electrochemical	3-NT: 0.002 µM 4-NQO: 0.001 µM	[36]
4	Glucose	Salt-facilitated plasma separation	Whole human blood	Whatman No. 4 filter paper	Wax printing	NA	Colorimetric	NA	[37]
5	Chymotripsinogen	MIP-facilitated SPE	Urine	Whatman No. 1 filter paper	Wax printing	Manual cutting	Fluorescence	0.34 µM by image analysis software 0.36 µM by naked eye observation 3.5 µM in real urine samples	[38]
6	SARS-CoV-2 nucleocapsid (N) protein	Centrifugal microfluidics	Human saliva	Whatman No. 4 filter paper	Wax printing	NA	Digital image colorimetry	10 pg/mL	[39]
7	Lactate, Na + and K +	MIP-facilitated solid-phase microextraction (SPME)	Saliva	Filter paper	Direct machining using a CNC machine	NA	Electrochemical	Lactic acid: 0.01 mM Na + and K + : 1 mM	[40]
8	Cortisol	MIP-facilitated SPE	Human sweat	Whatman No.1 filter paper	Laser printing	Laser cutting	Electrochemical	1.0 pM	[41]
9	Glucose and lactic acid	Hollow microneedle extraction	Interstitial fluid	Whatman No.1 filter paper	Laser engraving	Laser cutting	Digital image colorimetry	Glucose: 0.05 mM Lactic acid: 0.02 mM	[42]
10	DNA from live and dead bacterial cells, *E. coli* O157:H7 and *Salmonella spp.*	Solid-phase extraction (SPE)	Live and dead bacteria mixture	Cellulose paper and Whatman filter paper	Direct patterning using a CNC miller	NA	Colorimetric	25 CFU/mL	[43]
11	Lactose	Protein precipitation	Milk	Whatman No.1 filter paper	Wax printing	Possibly manual cutting	Electrochemical	0.14 mM	[44]
12	Copper	Paper-based soil-liquid extraction	Soil water	Whatman No. 1 filter paper	Atom stamp printing	Laser cutting	Colorimetric and distance-based	Cu2 + : 1 mg/L	[45]
13	Copper	Electromembrane extraction (EME)	Tap water, spring water, and anemic blood	Whatman No. 1 filter paper	Laser printing with toner ink	Laser cutting	Digital image colorimetry	3D origami: 20 µg/L	[46]
14	Mercury and lead	IIP-facilitated SPE	River and seawater	Whatman chromatography No. 1 paper, cotton tissue	Wax inkjet printing	NA	Fluorescence	Hg2 + : 0.18 µg/L Pb2 + : 0.07 µg/L	[47]
15	Nitrophenol isomer	MIP-facilitated SPE	Tap water	Whatman glass fiber paper	Wax printing	NA	Fluorescence	2-NP: 0.347 µM 3-NP: 0.303 µM 4-NP: 0.388 µM	[48]
16	Pesticide residues: chlorpyrifos, phoxim, carbaryl, triazophos, carbofuran and methamidophos	Organic solvent extraction	Head lettuce, water, tea and apple juice	Whatman No. 1 filter paper	Wax printing	NA	Colorimetric	Chlorpyrifos: 0.77 mg/L Phoxim: 0.39 mg/L Carbaryl: 0.25 mg/L Triazophos: 1.29 mg/L Carbofuran: 0.006 mg/L Methamidophos: 1.39 mg/L	[49]
17	Butachlor	MIP-facilitated SPE	Mung beans	Whatman No.1 filter paper	Wax printing	NA	Digital image colorimetry	1.43 ng/g	[50]
18	2,4-dichlorophenoxyacetic acid (2,4-D)	MIP-facilitated SPE	Cucumber	Whatman No.1 filter paper	Wax printing	Possibly manual cutting	Fluorescence	0.17 µM	[51]
19	Lead	IIP-facilitated SPE	Green tea leaves	Whatman Filter Paper No. 1	NA	Laser cutting	Digital image colorimetry	0.275 µg/mL	[52]
20	Streptomycin	MIP-facilitated SPE	Milk and human serum	Filter paper	Wax Printing	Possibly manual cutting	Electrochemical	0.9 pM	[53]
21	Naproxen	CMIP-facilitated SPME	Human serum	Whatman Grade 41 ashless filter paper	NA	Possibly manual cutting	Electrochemical	2.0 × 10^−7^ M	[54]
22	H5N1 virus	MIP-facilitated SPE	Human serum	Whatman No.1 filter paper	Wax printing	Laser cutting	Fluorescence	0.58 fM	[55]
23	Lead	Salting out	Urine	Whatman No. 1 filter paper	Laser printing using toner ink	NA	Electrochemical	9 µg/L	[56]
24	*E. coli*	Acoustofluidics-driven homogenization and filtration	Raspberry and strawberry	PDMS	Soft lithography	NA	Fluorescence	5 CFU/g	[57]
25	Sorbic acid	Micro-distillation	Food samples	PMMA	NA	Laser cutting	Colorimetric	NA	[58]

### Biomarkers

The NIH defines biomarkers as “a characteristic that is objectively measured and evaluated as an indication of normal biologic processes, pathogenic processes or pharmacological responses to therapeutic intervention.” [59] Although the exact definition of this term has shifted dynamically, biomarkers can be broadly defined as an indicator of the body’s biological state, specifically the onset of disease, progression, and prognosis [60]. They are better than conventional disease monitors because of their higher accuracy and shorter response time, allowing for more efficient clinical trials while also avoiding the associated ethical problems [61]. As a result, biomarkers are usually used for screening, characterizing, and prognosis in personalized medicine and other clinical applications. They are also used as drug targets in drug discovery and development sectors [60, 61]. They can be sampled from a wide range of samples including biological, environmental, and food-based. Until recently, conventional detection techniques like LC–MS/MS were used frequently for their detection. However, as mentioned previously, due to their several disadvantages, researchers pivoted to µPAEDs. In this section, the latest technological advancements in this field are discussed at length.

### Blood

Blood is composed of water, RBCs, WBCs, platelets, fibrinogen and other clotting factors, and proteins like albumin, glucose and electrolytes [62]. It is highly susceptible to change in composition due to various factors like disease, dehydration, and interindividual variability [62]. Blood sampling is less invasive when compared to sampling of tissue fluid and is the most accessible body fluid in clinical settings [63–65]. Although blood sampling is prevalent, plasma and serum are used more commonly [63, 65]. Plasma contains platelets and clotting factors and is usually stored to avoid coagulation. In contrast, serum is coagulated before storage to remove platelets and coagulation factors. It is usually used to avoid interference from other components [62]. The commonly found biomarkers in blood include glucose, omega fatty acids, albumin, and ascorbic acid [66].

Ascorbic acid (AA), also called vitamin C, is the most abundant and essential hydrophilic vitamin with antioxidant properties [34, 67–69]. But imbalanced concentrations of AA in blood can lead to oxidative stress, scurvy, neurological, cardiovascular, renal, and hepatic diseases and even cancer [34, 68, 69]. Thus, for its facile detection, Gautam et al. designed a reusable, durable, and eco-friendly µPAED integrating solid-phase extraction (SPE) with electrochemical detection. Filter paper and membrane strips were used to enhance sample flowability and on-line plasma extraction. Through hydrothermal synthesis, magnesium ferrite nanomaterials (n-MgF) were prepared. Consequently, it was dropcast onto the surface of the working electrode. Cyclic voltammetry (CV) and differential phase voltammetry (DPV) were used to detect and monitor AA in blood samples. Gautam et al. found that n-MgF improved the electron transfer and enhanced the device’s sensitivity. Upon further testing, they found that the device exhibited high stability even after 55 days of use and could be reused up to 14 times, with RSD values less than 2%. This biosensor was then successfully used for detection of AA in real samples. This µPAED is affordable, selective, and sensitive and can be applied in clinical settings (LOD 2.44 µM) [34]. However, it may prove difficult to operate in field-testing applications as potentiostats are required for electrochemical measurements. Although portable potentiostats are available, they are very expensive. Instead, an inexpensive and portable battery system integrated with smartphones can be used. Furthermore, digital image colorimetry can be developed and conjugated with this µPAED to enhance its accessibility.

In addition to AA, carcinoembryonic antigen (CEA) has also been found in blood. CEA is a stable, macromolecular, cell-surface glycoprotein. However, it is non-specific as it is used as an indicator for gastrointestinal, breast, pancreatic, liver, ovarian, and lung cancers and various non-cancer conditions like inflammation [70–73]. Detection of CEA is often challenging due to reduced sample concentrations and interference in complex matrices. To resolve these problems, Qi et al. designed an affordable µPAED integrating molecularly imprinted polymer (MIP)-facilitated SPE and electrochemical detection. The pattern and electrodes were printed onto filter paper through wax printing and manual brushing respectively. Using origami method, a unique and novel 3D-µPAED design was created which included movable valves, washing channels, and an electrochemical setup. MIPs were prepared using CEA and dopamine as the template and functional monomer respectively and deposited onto µPAEDs. CV was used to initiate the electropolymerization process before sample addition and was followed by a washing step. This removes non-specific templates and interactions and renders them with CEA-specific cavities. Once CEA binds with the MIPs, there is a change in the electrochemical properties which is then quantified to retrieve the concentration of CEA within sample. This device is affordable and has high selectivity and sensitivity (LOD 0.32 ng/mL) with the results comparable to ELISA [35]. It is a label-free, non-toxic alternative to conventional ELISA and involves simplistic operational procedures. However, proper washing must be performed to avoid incomplete cavity formation, false positive results, and reduced sensitivity. Additionally, screen printing can be used to print electrodes to ensure smooth and even surface.

Amatatongchai et al. developed a novel µPAED for the simultaneous electrochemical detection of two oncogenic oxidative stress biomarkers 3-nitrotyrosine (3-NT) and 4-nitroquinoline-N-oxide (4-NQO) using dual MIPs. The µPAED consisted of spherical reservoirs, microchannels, and screen-printed electrodes. The working electrode was coated with dual MIPs grafted onto GQDs (graphene quantum dots) capped with AuNPs. GQDs were synthesized from citric acid and were used to enhance the sensitivity of the device. To this, HAuCl_4_ and DI water was added and heated to form AuNP capped GQDs. Consequently, MPTMS was added to introduce thiol groups to this composite. Then, MIPs were formed using 3-NT and 4-NQO as templates. These templates were washed away with a mixture of methanol and acetic acid, leaving behind analyte-specific cavities. Square wave voltammetry (SWV) with oxidation peak potential of + 0.78 V and − 0.10 V was used to monitor 3-NT and 4-NQO respectively. The device showcased high sensitivity, selectivity, and specificity even in the presence of structural analogs of the selected biomarkers (LOD: 3-NT, 0.002 µM; 4-NQO, 0.001 µM) [36]. However, MIP preparation is quite laborious as incomplete washing greatly affects the device’s specificity. Additionally, portability may cause issues due to usage of potentiostats. Instead, in the future, digital image colorimetry (DIC) can be developed to resolve this problem and increase its accessibility. Despite these issues, this µPAED successfully and accurately detected 3-NT and 4-NQO in serum, whole blood, and urine samples.

Nilghaz et al. developed a simple µPAED combining salt-facilitated plasma separation with paper-based microfluidics. Briefly, wax printing was used to graft the pattern onto filter paper. Then, saline solution and colorimetric indicator for glucose were deposited onto the sample inlet and detection zones, respectively. A whole blood sample was then deposited onto the sample inlet zone. The highly saline environment creates high osmotic pressure which causes a shift in the shape of RBCs. These changed RBCs aggregate on the paper, while the plasma dissolves the salt resulting in a wicking effect, causing the migration of plasma onto the detection zone. A simple glucose determination test was conducted on the µPAED to determine if proper separation has taken place. The colorimetric glucose indicator changes color to green in the presence of glucose, which is found in plasma. A glucometer was used to quantify the results. Although other alternatives for this type of paper exist in the market, they are often expensive as highlighted by Nilghaz et al. In contrast, this device is affordable. By utilizing a simple separation technique, efficient and rapid plasma separation could be achieved, showcasing the device’s simplicity [37]. Despite its accessible and user-friendly approach, the shelf-life of the µPAED and its durability across various environments is unmentioned. Thus, further investigation into this aspect must be carried out. Additionally, although this device showcased the feasibility of simple, yet efficient plasma separation, there is no mention of LOD. Thus, further optimization and validation are necessary.

### Urine

Urine is the product of excretion which is an important and necessary biological function of the urinary system [74, 75]. The kidneys have a fundamental role in excretion as they concentrate some metabolites during the process. Therefore, although the bio-component profile of both urine and blood is similar, the concentration of these components is significantly higher in urine than in blood. Urine is a good measure for therapeutic response and diet monitoring since kidneys are also involved in the filtration of drugs and other contaminants from blood [74]. Unlike blood, plasma, or serum, urine is sampled non-invasively and can be collected in large volumes without causing distress to patients [75]. Other advantages of urine sampling include easy storage and transport, continuous monitoring, real-time and remote monitoring, and rapid testing [74, 75]. Also, urine is a great source of biomarkers due to its more stable and simpler proteome [76]. Some examples include hormones, oncogenic gene fragments, pluripotent stem cells, annexin-3, sarcosine, lactate, uric acid, glucose, ions, adenosine, small molecules, nucleic acids, neurotransmitters, and trace amounts of drugs [76–78]. These biomarkers have been linked to acute stress, chronic fatigue, prostate cancer, PTSD, anxiety, and drug abuse [78].

Rypar et al. consolidated MIP-facilitated SPE and distance-based fluorometry into a µPAED for the detection of chymotripsinogen (chymo), a model protein, in urine samples. Wax printing was used to print a pattern consisting of a measuring scale and sample, detection, and absorbent zones onto filter paper. Then, through self-polymerization, chymo-templated MIPs were prepared. Pre-polymerized MIPs were then deposited onto the test zone in µPAED, followed by polymerization, washing, drying, and blocking. Spiked urine samples were deposited onto the sample zone and transported to the detection zone via capillary action. After drying, fluorescamine was sprayed onto the detection zone, begetting immediate interaction with amino groups of the analyte. The µPAED was then placed into a dark box equipped with a UV lamp and smartphone to quantify the results, although instrument-free readout was also feasible. This device performed remarkably well in real samples, with high sensitivity and moderate selectivity even in the presence of interfering components (LOD 3.5 µM) [38]. However, in the presence of salts, moderate accuracy was observed. This could be attributed to a change in the ionic strength of urine, which affected the interaction between MIPs and chymo. Another issue they might face with repeated use is photobleaching and fluorescence quenching due to interference with other components in real samples.

### Saliva

Saliva is a slightly acidic bodily fluid secreted by a combination of major and minor salivary glands located in submandibular and periauricular regions [79, 80]. It contains water, enzymes like amylase and lipase, electrolytes, antibodies, leucocytes, proteins, cell fragments, food remnants, and microorganisms. The exocrine fluid protects the mouth using antibacterial enzymes, maintains teeth and oral mucosa, facilitates speech and eating, and hydrates the mouth [79–81]. Additionally, it is collected non-invasively and is suitable for patients who are uncooperative when taking blood or urine samples (like young children and elderly patients) [79, 81]. Furthermore, it is cost-effective with simple storage and transport and offers real-time analysis, home collection and screening, minimal cross-contamination, and safety for both health workers and patients [80–82]. Due to the juxtaposition of salivary glands with blood vessels, the mouth and thereby saliva is an abundant repository of biomarkers [80]. Some examples include ammonia, thiocyanate, nitrite, lactate, glucose, aldehydes, viruses like SARS CoV-2, hormones, nucleic acids, electrolytes, lactoferrin, cortisol, and proteins [79, 81]. These biomarkers have been associated with oral cancer, Alzheimer’s disease, Huntington’s disease, Cushing’s syndrome, and Sjögren’s syndrome [80, 82].

In this light, Liu et al. developed a rapid testing µPAED for instrument-free detection of SARS-CoV-2 nucleocapsid (N) protein involving centrifugal microfluidics and colorimetric detection. The pattern was printed using wax printing and consisted of sample, test, and control zones connected via microchannels. The test and control zones were pre-deposited with poly-L-lysine (PLL) for stable antibody attachment, and the µPAEDs were treated with antibodies following an incubation step. To extract the protein biomarker, Liu et al. used a centrifugal microfluidics platform composed of a DC motor, controller, and charge supply. For colorimetric detection of N protein, they used AuNPs. AuNPs were prepared, mixed with the sample solution, and pipetted onto the sample zone of µPAED. On reaching the test zone, the AuNP-labelled N-protein binds stably to the antibodies, resulting in a color change to red. An image was captured, and color intensity was analyzed using imaging software, where they found that red color intensity directly correlated with N-protein concentration. This device exhibited a large linear range (10–1000 pM) and low LOD signifying its high sensitivity (LOD 10 pM) and avoided the usage of external pumps and complex instruments by incorporating centrifugal microfluidics [39]. It was successfully applied for extraction and detection of N-protein in human saliva samples, with results equivalent to LFA strips. Despite its high specificity, the adoption of antibodies significantly increases the device’s overall costs. Alternatively, MIPs can be used to maintain specificity and reduce costs. Also, multiplexed analysis is unachievable with this device. This aspect can be improved upon with the usage of dual or hybrid MIPs.

Zheng et al. also designed a self-driven µPAED for electrochemical detection of lactate and electrolytes (Na^+^ & K^+^) in saliva using solid-phase microextraction (SPME). The design consisted of a liquid self-actuated component, MIP-monolithic column encapsulated within the microchannel, capillary pump, and an electrochemical zone for detection. The monolithic column was designed with a specific lactate capture capacity using MIPs and was used for extraction. Sample was deposited into this column and rinsed with acetonitrile (ACN) to remove impurities and non-specific interactions. Finally, lactic acid was eluted out of the column and transported to the electrochemical zone. Three electrodes specific for Na^+^, K^+^, and lactic acid were present at this zone. The interaction between analyte and its respective electrode caused a change in electrochemical properties which manifested as a change in impedance. This was quantified to determine their concentration in sample. This device exhibits high specificity and sensitivity (LOD: lactate, 0.01 mM; Na^+^ and K^+^, 1 mM), offers simultaneous analysis, and is user-friendly [40]. But electrochemical detection is not feasible for field-testing. Instead, DIC can be developed for instrument-free read out, as seen in Liu et al.’s study. In addition, MIPs can lose their efficiency after several uses. To prevent this, MIPs can be combined with other markers like aptamers to enhance both efficiency and stability.

### Sweat

Sweat is mostly secreted by eccrine sweat glands which are profusely present throughout the body. Production of sweat normally occurs due to increased body temperature and exercise, bestowing sweat with thermoregulatory properties [83]. Also, it maintains the balance of electrolytes in the body by reabsorption and acts as an immunity barrier for the skin due to its slightly acidic pH [83, 84]. Sweat secretions by eccrine glands contain mostly water, NaCl, micronutrients, electrolytes, metabolites like ammonia, urea, ethanol, glucose, and lactate, along with cytokines, stress hormones like cortisol, and trace elements like iron, zinc, and copper [83–86]. Sampling sweat from individuals is much easier than blood or urine due to its non-invasive nature and wide distribution in the body, facilitating continuous and real-time monitoring [83, 84, 86]. It also offers a decentralized approach, making it possible for bedside testing and diagnosis [84, 86].

**Fig. 2:**
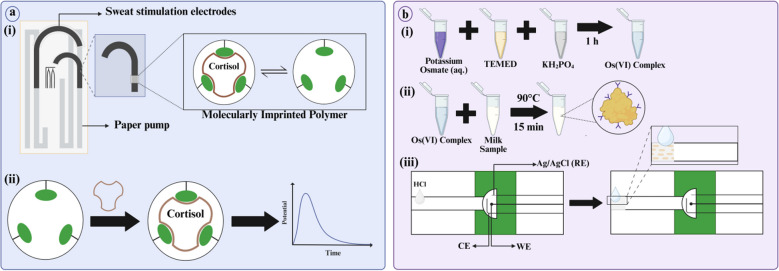
**a** (i): Depicts the final µPAED and grafting of MIPs onto the electrode surface. MIPs were prepared using cortisol (brown) as the template molecule and APTES (green lobes) as the functional monomer. After polymerization and blocking, the template was washed away. This leaves behind a cavity specific to cortisol in the MIP. (ii): When cortisol (brown) interacts with these empty cavities, it results in a change in impedance which has been represented by a simple graph in the figure (adapted from Ref. [87]) **b** (i): The overall process of formation of the labeling complex Osmium (VI) has been described. (ii): Os(VI) complex is mixed with milk samples and heated at 90 °C for 15 min to complete the lactose labelling protocol. Lactose (yellow) is labelled with the complex (Y-shaped in purple color). (iii): The detection and extraction process has been highlighted. After µPAED fabrication, HCl was pre-deposited onto the sample zone. Due to the acidification of proteins by HCl, milk proteins (light orangish-yellow) precipitate out. In this way, the interfering components are removed. Then the milk sample is transported to the detection zone with the 3-electrode set-up for electrochemical detection (not shown here) (adapted from Ref. [44]) (created in https://biorender.com/)

To realize this, Garg et al. developed a wearable sensor using MIPs and an iontophoresis module for continuous electrochemical detection of cortisol in human sweat samples, a biomarker of chronic fatigue and acute stress (Fig. 2a) [78, 87]. Laser printing and cutting were used to fabricate the µPAED. Then, MIPs were prepared using cortisol and APTES as the template and functional monomer respectively. Consequently, laser-induced graphene (LIG) electrodes were electropolymerized with prepared MIPs, and electrochemical impedance spectroscopy (EIS) was used for detection. When cortisol binds to MIPs, a change in impedance is recorded and quantified to identify its concentration. This wearable sensor was used by humans performing cycling, demonstrating its durability. Picomolar LOD (1 pM) indicates its high sensitivity, and the involvement of MIPs in the design greatly enhanced its specificity [87]. This µPAED is portable and self-cleaning, offering label-free detection, noninvasive sampling, and real-time analysis of cortisol in sweat. However, the secretion rate of sweat differs on an interindividual basis, though this can be tweaked by adjusting iontophoresis module settings. For continuous monitoring, the iontophoresis module requires a continuous supply of voltage, which may irritate the skin [41]. Also, as mentioned previously, MIPs may lose their efficiency after multiple uses. To avoid this, they can be prepared with aptamers. In addition, this device cannot perform multiplexed analysis yet, which can be improved by incorporating dual and/or hybrid MIPs.

### Interstitial fluid

Interstitial fluid (ISF) constitutes 75% of extracellular fluid (ECF) and is mostly found in dermal skin [41, 83, 88]. Since the dermal layer is highly perfused with blood vessels, there is a high rate of fluid and component exchange between blood and ISF, resulting in similar analyte concentrations [41, 89]. In fact, ISF is often preferred over blood as it is free from clotting factors and other constituents like RBCs and leucocytes. Furthermore, it contains higher concentrations of analytes and can be sampled in large volumes [88, 89]. This clear biological fluid contains proteins, lipids, waste substances, electrolytes, glucose, lactate, cortisol, urea, insulin, hemoglobin, cytokines, albumin, and immunoglobulins [41, 83, 89, 90]. ISF is usually extracted through capillary wicking, suction by vacuum, microdialysis, reverse iontophoresis (RI), and microneedles. Among these, RI and microneedles are preferred as they produce stable biomarker fingerprints through minimal skin penetration [41, 83, 88, 89]. However, ISF sampling also poses some problems. Sample extraction through skin puncturing may alter analyte concentration [41]. Other problems include delayed ISF replenishment rate, time-consuming sample withdrawal and extraction, and low ISF availability on skin. Together, these issues are unfavorable for long-term and continuous monitoring [41, 83].

To resolve these issues, Cheng et al. designed an affordable and user-friendly µPAED incorporating hollow microneedle (HMN) extraction and DIC for the detection of glucose and lactic acid in ISF. The design consisted of two channels, dual spherical detection zones and a rectangular component. The two detection zones were pre-deposited with 4-AAP, TOPS, and HRP solutions in addition to glucose oxidase and lactate oxidase. HMNs and µPAED were fixed onto PDMS backing forming the bottom layer. The top layer consisted of a single cavity for negative pressure production and quick, pain-free sampling without external pumping. Cheng et al. observed no visible color change after reaction between analyte and the reagents in detection zone. Thus, images were captured using smartphone and analyzed using an image analysis app. The sensor showcased high sensitivity (LOD: glucose, 0.05 mM; lactic acid, 0.02 mM) and high selectivity [42]. It is rapid, portable, and eco-friendly; offers simultaneous analysis; and shows potential for a wearable sensor. Despite this, the sensor was not tested on human skin. Furthermore, the device’s operation may be affected by interindividual variations in skin perforation and pigmentation. Thus, further investigation into these areas is required.

### Other samples

Raw milk and food like lettuce contain pathogenic bacteria like *E. coli* (O157:H7), *Salmonella* spp., *Campylobacter* spp., and *Listeria monocytogenes*. These microbes cause harmful effects on human health, resulting in infections, gastroenteritis, bloody diarrhea, hemolytic uremic syndrome, permanent organ failure, and in worst cases, death [91–93]. Of them, *E. coli* (O157:H7) and *Salmonella* spp. are the most commonly identified bacteria [92]. Trieu et al. designed an all-in-one µPAED integrating SPE, loop-mediated isothermal amplification (LAMP), and colorimetry for the facile detection of DNA isolated from these bacteria. The µPAED consisted of 2 parts. The first part, called the splitting pad, contained a central reservoir connected through microchannels to four peripheral reservoirs. The second part contained purification, reaction, dye, and wicking pads. Chitosan-coated and methylene blue (MB)-coated paper discs constituted the purification and reaction pads, respectively. Additionally, the reaction pad contained the reagents required for LAMP. The device was completed by folding the first part onto the second part. Extraction of bacterial DNA occurs through electrostatic interactions between DNA and chitosan. Following a washing step and device completion, the extracted DNA is transferred to the reaction pad where it reacts with the LAMP reagents. To complete this reaction, the device was heated. The resulting DNA amplicons interact with MB, resulting in a color change from colorless to blue. This integrated all-in-one µPAED demonstrates high specificity (because of the primers) and sensitivity (LOD 25 CFU/mL) [43]. It is affordable, user-friendly, rapid, eco-friendly, and versatile and offers multiplexed detection. However, the usage of specific primers may impact its effectiveness against all pathogens. Instead, universal primers can be used. Additionally, analyte concentrations lower than LOD may not be detected in environmental and food samples.

Nilgaz et al. designed an acoustofluidic polydimethylsiloxane (PDMS)-based device for homogenization and filtration of samples prior to detection, overcoming the need for off-chip sample preparation. First, a master mold for the chip was designed using soft lithography. After casting and curing PDMS, holes for inlet and outlet zones were punched. A glass slide was used as backing for PDMS, and an acoustic transducer was epoxy-glued onto it. The transducer was connected to a generator and amplifier. Then, hydrophilic yarn was inserted into the outlet. The resulting device contained two zones, a microvortexing zone for focusing acoustic energy and producing turbulent flow and a filtration zone consisting of micropillars for trapping the debris effectively. To realize on-chip sample preparation, spiked samples of fresh fruit were introduced into the inlet. Through the generation and amplification of vibrations, a vortical flow was generated in the device facilitating sample mixing and homogenization. Following filtration by micropillars and the porous yarn, the samples were carried through wicking to the outlet for collection. DNA extraction was conducted, and qPCR was used for their quantification using real-time fluorescence. This innovative device is portable, rapid, affordable, and sensitive. Its utilization of acoustic transducer for homogenization eliminates the need for external pumping (LOD 5 CFU/g) [57]. But it is also dependent on external electronics like transducer, generator, and amplifier for its operation. Additionally, its efficiency in highly viscous samples like syrups and honey has not been verified. Furthermore, the device is not truly integrated as DNA extraction and detection was carried off-chip, which was not the case with Trieu et al.’s device. To overcome these challenges, this device can be incorporated with µPADs to realize on-chip sample preparation and extraction, similar to Trieu et al.

Dortez et al. designed a µPAD using a simple extraction approach—protein precipitation—for the detection of lactose from milk samples (Fig. 2b). Lactose is a disaccharide comprising galactose and glucose. It promotes the growth of healthy gut microbes, facilitates mineral absorption, and supports the immune system [94]. In their design, wax printing was used to print the design onto filter paper, and screen-printing was used to print a three-electrode system onto µPAD. For the extraction, a small volume of HCl was deposited onto the sample zone and dried. Upon the addition of milk samples, protein precipitation was observed, which effectively removes other interfering agents. For facile detection, the lactose molecules were labelled with Osmium(VI) complex via mixing and heating. SWV was performed, and an oxidation peak of lactose-Os(VI) complex at − 0.75 V was recorded and used for qualitative analysis. In this fashion, different milk samples were tested, and detection was successful in all. The RSD values for precision and reproducibility indicate that the device is highly precise and reproducible. The device is affordable, accurate, rapid, disposable, and sensitive, requiring no external reagents [44]. Although the LOD is low (LOD 0.14 mM), it is not comparable to HPLC and can only be used for screening purposes. Processes like sample addition and electrochemical detection are not fully automated and may require experienced handling, which is not user-friendly. Also, electrochemical detection relies on potentiostats which are not suitable for field testing. Although portable potentiostats are available, they are very expensive. Instead of relying on instruments for retrieving the results, digital image colorimetry can be used as an alternative.

### Chemical contaminants

Abnormal concentrations of chemicals detected beyond their usual habitat are categorized as chemical contaminants. When these become potent enough to affect humans and ecosystems, they are called chemical pollutants. Nonetheless, exposure to these chemicals is harmful as they do not degrade easily. Due to their persistent nature, they can accumulate within the body causing detrimental effects like cancer [18, 95]. These chemicals have been found in water, aquatic ecosystems, food products, and biological fluids. In the subsequent sections, recent advances in development of µPAEDs for detection of various chemical contaminants in these fluids have been discussed.

### Water

Water is the most essential solvent for humans and the environment. Clean water, free from toxins and chemicals, is crucial for their welfare. Moreover, it is used in food, electronics, and pharmaceutical industries. However, these industries are also responsible for water pollution. Due to anthropogenic, domestic, agricultural, and industrial activities, harmful chemicals like heavy metals, organic matter, biocides, plastics, and pesticides are released into local water bodies and thus greatly contribute to water pollution [96, 97]. Heavy metals are especially harmful for both the environment and humans. Since they are not biodegradable, they tend to accumulate in various organisms on the food chain and ultimately compromise human health. Most heavy metals are highly toxic to humans and can cause various organ damage, cancer, cardiovascular diseases, impaired fertility, neurological disruptions, and gastrointestinal issues [96–99]. Some examples of heavy metals include copper, mercury, and lead.

**Fig. 3:**
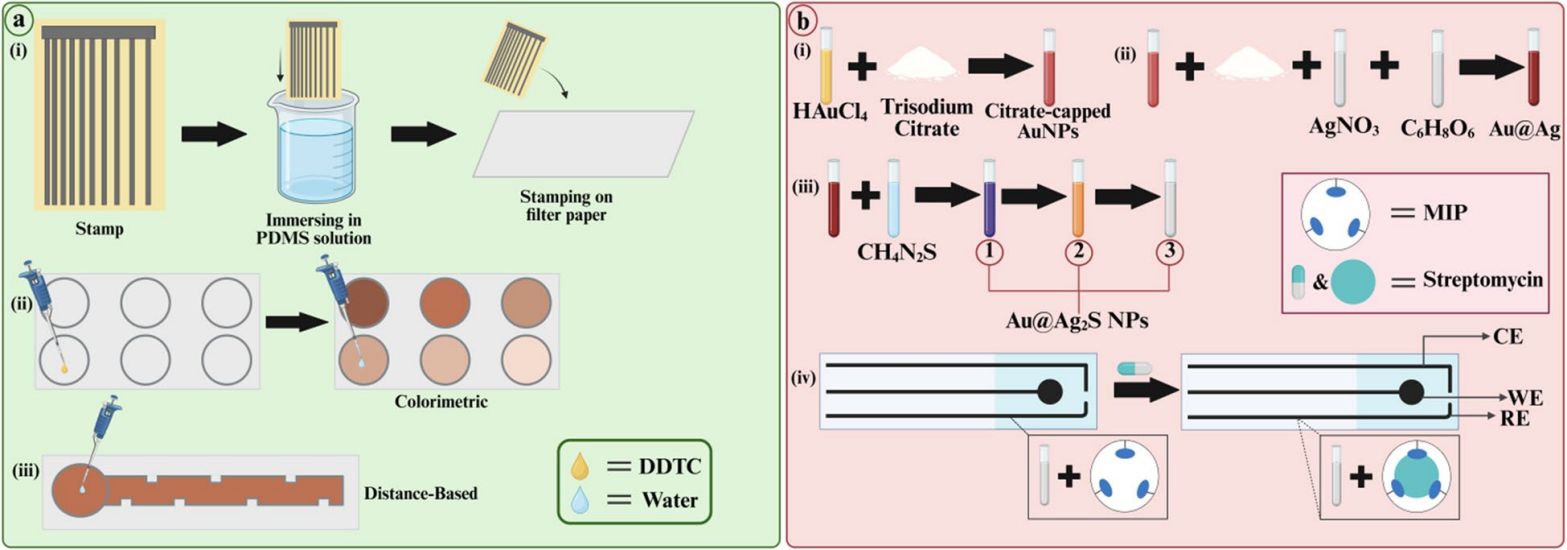
**a** (i) Depicts the novel stamp designed by Guan et al. for the fabrication of µPAED. The design was engraved onto a sponge using a laser engraving machine. The sponge or atom stamp was immersed in PDMS solvent. Afterwards, this stamp was used to print the design on filter paper. (ii) Filter paper fabricated via atom stamp printing was cut into circular discs. Then yellow-colored DDTC reagent was pre-deposited onto these discs. In water samples with Cu^2+^, there is an interaction between copper ions and DDTC forming a brown complex in weak alkaline pH. Through these discs, colorimetric detection was carried out. (iii) Similarly, µPAEDs fabricated via atom stamp printing were used for a distance-based assay as well. Here also, DDTC was pre-deposited on the sample zone (adapted from Ref. [45]. **b** (i) Describes the preparation of AuNPs (red). (ii) These AuNPs were mixed with trisodium citrate (white powder), silver nitrate, and ascorbic acid solutions forming Au@Ag core shells (dark red). (iii) After the addition of thiourea to these core shells, Au@Ag_2_S-NPs were formed following a series of color changes (purplish-red (1) to orange (2) to colorless (3)). (iv) MIPs were formed with streptomycin (pill-shape) and acrylamide (blue lobes) as templates and functional monomers. Then, a mixture of the NPs and MIPs was pre-deposited onto the detection zone on µPAED. Only in the presence of streptomycin is a change in electrochemical properties recorded (adapted from Ref. [53]) (created in https://biorender.com/)

Guan et al. designed a unique paper-based soil-liquid extraction (PSE) device, integrating it with 3D-µPADs for detection of copper (Cu^2+^) in soil water (Fig. 3a). Copper affects the gastrointestinal tract causing nausea, vomiting, spasms, and stomach cramps [97, 98]. Using atom stamp printing, they imprinted the pattern onto paper using a sponge. The novel paper–based extraction device was developed by alternative stacking and gluing of µPADs and PDMS films. The resulting 3D-µPAED was divided into two storage units and integrated with a custom setup for PSE, omitting the filtration step and thus enhancing the accuracy of the overall process. Circular patches of filter paper were immersed in DDTC solution. When Cu^2+^ reacts with DDTC, a brownish-yellow complex is formed. Guan et al. found that the concentrations recovered using µPAED were similar to those recovered by conventional techniques. They also found that the extraction time was shortened to 40 min due to the use of a micropump for continuous circulation of the solvent (LOD 1 mg/L) [45]. With this novel µPAED, the highest resolution and accuracy (95–98%) can be achieved within short time period, as compared to the long time taken through conventional extraction techniques. It is also affordable, reusable, and portable, with adjustable operational size. However, simultaneous analysis with this device is not yet possible, and efforts should be made in this area to enhance its versatility.

Another such µPAED integrating electromembrane extraction (EME) and DIC was developed by Alidoust et al. Unlike Guan et al., they used a prevalent extraction technique for copper extraction from tap water, spring water and even anemic blood samples. The 3D-µPAED was created by assembling four layers with the first three layers being sample loading-cum-extraction layers and the bottom layer as the detection layer, separated by an NPOE-coated polypropylene sheet. Electrode sheets were adhered to the topmost and bottommost layers. To complete the device, it was folded. After sample deposition to the topmost layer, electric potential was applied on µPAED, causing sample dispersion through several layers and thereby facilitating copper extraction. Upon reaching the detection zone, a mixture of NCP and HACHA was deposited. An image of the detection zone was captured after drying for further analysis. The device successfully and accurately detected copper in all samples. It showed high sensitivity (LOD 20 µg/L) and selectivity. Moreover, it requires less power and is user-friendly [46]. However, pH adjustments were performed prior to extraction and detection. This limits this µPAED’s working as such devices may be unavailable in remote areas.

On the other hand, Wang et al. developed a rotatable hybrid microfluidic device which integrated ion imprinting technology (IIP) and fluorometry for the detection of mercury (Hg(II)) and lead (Pb(II)) in river and seawater samples. These heavy metals damage kidneys, liver, and respiratory system and may also result in miscarriage, stillbirth, coma, convulsions, mitochondrial impairment, chest pain, and shortness of breath [97–99]. The hybrid µPAED was made from cloth and paper and contained three layers. The top layer had four channels with dual branches for sample zones. The middle wax layer contained eight square cut-outs positioned below the sample zones. The bottom layer had four channels for each analyte, and their ends were glued to IIP-coated cloth pieces. To make the IIP-coated cloth pieces, the cloth was first treated with HCl, APTES, and Hg(II)/Pb(II) mixture. Then, TGA-modified CdTe-QDs were prepared via sol–gel method, grafted onto treated cloth surface, followed by polymerization, blocking, and washing. The 3D-µPAED was rotated clockwise and anti-clockwise for Pb(II) and Hg(II) detection respectively. IIPs act similarly to MIPs as they also have cavities specific towards the analyte and its functional groups. Target ions bind specifically to these cavities, thus getting extracted from the sample (recovery: Hg(II), 96.2–1141; Pb(II), 97.7–105.4 across all samples). Upon binding to their respective IIPs, the analytes accept electrons from CdTe-QDs, which results in fluorescence quenching. This device was successfully applied to real water samples, showing high selectivity, specificity, and sensitivity (LOD: Hg(II), 0.18 µg/L; Pb(II), 0.07 µg/L) with results comparable to ICP-MS [47]. The combination of paper and cloth makes this device affordable and user-friendly while enhancing the extraction efficiency without external filtration or pumping units. This approach helps to overcome the need for centralized laboratory testing, making it more accessible as compared to traditional methods. However, a spectrofluorometer is required to analyze the results. Alternatively, DIC can be used for instrument-free read out.

Similarly, Zhu et al. integrated MIPs and carbon quantum dots (CQDs) with µPAEDs for extraction, detection, and differentiation between nitrophenol isomers in tap water samples. These analytes can cause skin and eye irritation, kidney and liver damage, and cancer [100]. This device contained three layers. Using a hydrothermal method, CQDs were prepared and grafted onto filter paper. Following this, MIPs were prepared using the analytes (2-NP, 3-NP and 4-NP) as templates. After polymerization and washing, these MIPs were deposited onto CQD-modified paper forming the µPAED’s bottom layer. The middle layer contained a sample inlet, three detection zones, and microchannels. Finally, the top layer contained cutouts for sample and detection zones. The prepared MIPs possess cavities specific to the isomers. Thus, upon sample deposition, the isomers interact and bind specifically to these cavities resulting in their extraction from the sample. Fluorescence signals were used to detect the analytes. When the isomers bind to MIPs, they participate in electron transfer with CQDs, which causes fluorescence quenching. When these are removed from MIPs, the fluorescence emissions return to normal levels. The reduced emissions are quantified to retrieve their concentration. Zhu et al. were able to distinguish between the three isomers due to their unique and distinct emissions in tap water samples successfully. This µPAED is user-friendly, affordable, accurate (100%), highly selective, specific, and sensitive as is evident from the LODs for 2-nitrophenol, 3-nitrophenol, and 4-nitrophenol given as 0.347 µM, 0.303 µM, and 0.388 µM respectively [48]. However, it requires a spectrofluorometer for analysis making it unsuitable for field-testing. Although handheld spectrofluorometers are available, they are expensive. A suitable alternative for this is DIC.

### Food

Food contains all the nutrients and minerals required for growth, development, and well-being of human health. But, due to an increase in global agricultural demand, farmers have turned to using pesticides for enhancing crop yield. In fact, Lazarević-Pašti et al. found that vegetable production would decrease by 54% without their usage [101]. Although their benefits for crops are manifold, they result in harmful defects in humans, animals, and the ecosystem. Agricultural produce, like fruits and vegetables, and animal products, like milk, eggs, and meat, may contain pesticide residues which, when ingested by humans, may lead to skin irritation, headaches, dizziness, coma, confusion, seizures, reproductive problems, endocrine disruption, and heightened susceptibility to leukemia [101–103].

Jin et al. developed a rapid-testing µPAED integrated with organic solvent extraction for detection of pesticides in foods. A flower-shaped pattern with eight detection zones connected to a central reagent reservoir through microchannels was wax printed onto filter paper. Eight different solvents, IPA and AChE solution, were pre-deposited onto detection zones. ACN was used as an organic solvent for extraction from lettuce heads, tea, apple juice, and water samples. The extracts were deposited onto µPAED and allowed to evaporate, which extracted the pesticides. For colorimetric detection, AChE and IPA were added. When IPA interacts with AChE, it changes color (yellow to blue). But in the presence of pesticides, the color formed is less intense due to inhibition. After 7 min of reaction time, DIC was performed. Overall, this method enhances recovery of pesticides from samples and eliminates the risk of false negatives. Unlike conventional techniques, it does not require enzyme immobilization and allows for highly sensitive detection (LOD: chlorpyrifos, 0.77 mg/L; phoxim, 0.39 mg/L; carbaryl, 0.25 mg/L; triazophos, 1.29 mg/L; carbofuran, 0.006 mg/L; methamidophos, 1.39 mg/L) [49]. However, it is still susceptible to interference in more complex matrices. Also, it cannot extract highly polar pesticides from samples using ACN as the only solvent. Instead, a mixture of organic solvents can be used.

Wu et al. integrated MIPs with digital image colorimetry (DIC) for real-time detection of butachlor, a herbicide, in mung beans. Pre-polymerized MIPs synthesized from surface molecular imprinting technique with butachlor as the template were coated onto ZnFe_2_O_4_ surface. Following polymerization and washing, MIPs were deposited onto the µPAED’s reaction pads. Once the sample reaches the reaction pad, a mixture of TMB and H_2_O_2_ was added for detection. Color development depends on the Fenton reaction and interaction of TMB with OH^−^ ions. In the presence of butachlor, there is no Fenton reaction. However, in the absence of butachlor, hydrogen peroxide molecules pass through free cavities of MIPs and react with ZnFe_2_O_4_, releasing OH^−^ free radicals. TMB then reacts with these ions, forming blue colored oxTMB. Thus, in the presence of butachlor, the color changes from blue to colorless. Smartphones were used to capture the image and analyzed using image analysis software. The device performed extremely well with samples of mung beans with no interference, demonstrating its specificity and selectivity (recovery, 93.4–106.4%; RSD, less than 5.6%). However, it was unable to detect any butachlor in unspiked samples, which may be due to the absence of butachlor or concentrations lower than LOD (LOD, 1.43 ng/g) [50]. In addition, there is an elevated risk of false positives with MIPs as analyte’s structural analogs like alachlor may bind to the cavities [104].

Hao et al. also designed a similar device for the detection of 2,4-dichlorophenoxyacetic acid (2,4-D), a herbicide, in cucumbers but used fluorescence detection instead of DIC. Their final device consisted of three layers. The top layer contained microchannels, the middle layer contained MIP-fluorescent substrate conjugate, and the bottom layer contained MIP-sample mixture. The MIPs were prepared using 2,4-D and APTES as template and functional monomer respectively. Pre-polymerized MIPs were coated onto polyester fibers functionalized with CdTe-QDs, followed by polymerization and washing. The conjugate MIPs were deposited onto the detection zone in µPAEDs. On reaching this zone, the sample interacts with the conjugate MIPs. 2,4-D binds to the cavities and triggers fluorescence quenching through electron transfer at 650 nm. Hao et al. also used rhodamine B as a reference fluorophore and calculated the concentration of 2,4-D through ratiometric fluorescence. They reported that the device had a low LOD (LOD 0.17 µM) and large linear range, signifying its high sensitivity [51]. It is also highly selective and accurate, with a short response time of 10 min making it ideal for POC applications. However, its use in remote areas may be affected, as specialized equipment is needed for analysis. DIC is a suitable alternative, as it offers real-time analysis and instrument-free read-out.

In some foods like tea leaves and vegetables, heavy metal contamination has been found. These metals accrue in human bones and fatty tissues, causing neurological and cardiovascular diseases. In particular, lead is carcinogenic and has been proven to cause bone fractures, hypertension, and damage to liver, kidney, and lungs [103, 105]. In this regard, Lou et al. designed a lateral flow test strip for extraction-cum-detection of lead from dried green tea leaves. Nitrocellulose membrane and absorbent pad were fixed onto a PVC layer on the front and back ends respectively. Lead-specific IIPs were prepared from lead nitrate solution and polymerized. These IIPs were grafted onto filter paper. The modified filter paper was glued between nitrocellulose membrane and absorbent pad. These LFA-µPAED strips were added to a microwell plate followed by the addition of green tea extract. XO reagent was added to visualize change in color from light yellow to orangish-red. DIC was performed, enabling instrument-free read out and real-time detection (LOD 0.275 µg/mL [52]. This device is POC-friendly and can be operated in resource-limited settings. However, extreme care should be taken during the washing step as improper washing can affect the cavity structure which may affect specificity.

Wu et al. designed a novel µPAED for on-chip sample preparation and detection of sorbic acid, a food preservative, from various food samples ranging from beverages and sauces to condiments and pseudo-meats. First, a PMMA chip consisting of a mixing chamber, reagent chamber, and detection chamber was prepared via laser cutting. These chambers were bonded through thermocompression. Then, a micro-spectrometer, pneumatic system, and a micro-heater were integrated into the device. For the extraction of sorbic acid from food samples, micro-distillation was carried out. The extracted preservative was then added to the chip, where it was mixed with an oxidizing agent and TBA in the mixing chamber under pneumatic pressure. This was followed by heating via micro-heater to induce the colorimetric reaction, resulting in a color change to pink. This pink product flows to the detection chamber where the quantification occurs. Wu et al. also extracted and detected other contaminants such as benzoic acid, sulfur dioxide, and formaldehyde, showcasing the device's versatility. Additionally, the designed µPAED is affordable, portable, highly sensitive, and rapid, with results retrieved within 20 min [58]. Despite its versatility, the current device is incapable of simultaneous analysis as only individual analysis is possible. To overcome this, a multi-channel design can be incorporated.

Adampourezare et al. integrated MIPs and electrochemical analysis for the detection of streptomycin in milk and serum samples using µPAEDs (Fig. 3b). Streptomycin is an antibiotic isolated from *Streptomyces griseus* and has been used in tuberculosis treatment [106]. Local wax was used to print the pattern on filter paper, and a 3-electrode system was drawn using a pen and nanoink. Hydrophobic paper was adhered to the back of the device to prevent leakage. Then, MIPs were prepared using streptomycin and acrylamide as template and functional monomer respectively. After polymerization and washing, they were grafted onto the electrodes. Electrochemical analysis was carried out with CV and DPV, with peak oxidation potential at + 0.1 V. A drop in current was observed when streptomycin interacted with MIPs. The greater the drop in current, the higher the concentration of streptomycin in sample. The oxidation peak potential of this device is lower than other streptomycin detecting µPADs, which when combined with its large linear range (1 pM to 1 µM) and low LOD (0.9 pM) indicate its superior sensitivity [53]. This µPAED also shows high specificity due to MIPs and high stability and reproducibility due to low RSD values. However, potentiostat is needed for electrochemical analysis, which makes this device unsuitable for field-testing. Instead, DIC can be used. In addition, this device has only been used in serum which is free from interfering agents. Further investigation into its efficiency in other biofluids needs to be conducted. Furthermore, electrode surfaces are prone to fouling in complex matrices. To avoid this, anti-fouling agents can be used.

### Serum

When contaminated food and drinking water are ingested, they tend to accumulate in body tissues via bioaccumulation [102, 107]. Due to the equilibrium interaction between blood and these tissues, the chemicals can transfer into systemic circulation. Serum and, by extension, blood are reliable body fluids for monitoring applications as they reflect the true concentration of chemical contaminants within the body. These fluids have been used to analyze long-term exposure to various chemicals like pesticides and drugs and their effects on the body [108].

Haghgouei et al. designed an all-in-one µPAED integrated with conductive MIPs (CMIPs) and electrochemical analysis for the detection of naproxen, a non-steroidal anti-inflammatory drug (NSAID) in human serum samples. NSAIDs have been shown to cause gastrointestinal bleeding and ulcers, hypertension, edema, and nephrotoxicity manifesting as chronic kidney disease [109, 110]. Herein, polypyrrole (PPy) was deposited onto filter paper through chemical polymerization with anion dopant and oxidant. The addition of PPy makes the paper conductive and the µPAED more specific due to its interaction with the analyte. Through electropolymerization, naproxen-specific CMIPs were prepared and deposited onto the paper. The modified µPAED was then immersed in spiked serum samples, and electrochemical analysis was conducted. The oxidation peak potential was recorded at + 0.6 V. This device showed high accuracy and stability (RSD 5%) for up to 2 months. Large linear range and low LOD (LOD 2.0 × 10^−7^ M) suggest that the device is highly sensitive as well [54]. The prepared CMIPs were highly specific towards naproxen, with poor specificities for other drugs from the same family. But, electrodes are prone to fouling in more complex matrices and can greatly interfere with the sensitivity and accuracy of measurement. This can be rectified by coating them with anti-fouling agents.

Gong et al. designed a portable µPAED incorporating MIPs and fluorescence for the detection of avian influenza H5N1 virus in human serum samples, which is responsible for causing respiratory damage and severe systemic sepsis [111]. Here, MIPs were prepared using H5N1 as a template and polymerized on patterned filter paper. The template was washed away with a buffer solution. Zeolite imidazolate framework with rhodamine B was prepared and modified with aptamers specific for H5N1. The modified framework was grafted onto MIPs, forming the fluorescent probe. The binding of H5N1 with MIPs results in fluorescence quenching and a visible change of color. Thus, instrument-free qualitative analysis is possible within 60 min. The linear range and low LOD (LOD 0.58 s) signify its high sensitivity, and the utilization of MIPs enhances the µPAED’s specificity and selectivity [55]. This device is portable and offers dual detection. However, in remote areas, it can only be used for screening as specialized equipment is required for quantitative analysis. To make it more accessible and efficient, DIC can be used. In addition, this device has only been tested on human serum samples. Further investigation into its operational effectiveness and efficiency in other biological fluids needs to be conducted.

### Urine

After entering systemic circulation, chemical contaminants undergo metabolism in the liver. Then, they may be accumulated within the body inside tissues or eliminated through urine. Thus, by understanding, analyzing, and comparing plasma and urinary concentrations, a complete and detailed profile of these chemicals can be generated. For example, lead enters the body through inhalation and ingestion of contaminated air, food, and drinking water respectively. It is then metabolized in the liver by P-450 enzymes. Finally, it is eliminated from the body through urine gradually. However, even light exposure to lead may result in reproductive system disruption, sleep deprivation, anemia, vertigo, and migraines [97, 112].

In light of this, Wang et al. integrated a simple protein precipitation method for extraction of lead (Pb(II)) and removal of proteins from urine with electrochemical detection. The pattern was printed onto filter paper through wax printing. The paper was then cut into strips, each of which contained sample and detection zones. The sample zone was pre-deposited with ammonium sulfate. Proteins in urine interact with ammonium sulfate and precipitate due to salting-out effect. Then, the extracted sample is transported to the detection zone. Here, a 3-electrode system was set up using gold-plated PET electrode as working electrode, and anodic stripping square wave voltammetry (SWV) was performed with oxidation peak potential set to − 0.6 V. With a low LOD (LOD 9 µg/L), this µPAED demonstrates moderate sensitivity as concentrations lower than 9 µg/L cannot be detected [56]. Moreover, it is affordable and easy to use. But it may not be operational in remote areas as potentiostat is required for detection, and portable potentiostats can be expensive. Instead, digital image colorimetry (DIC) can be used for portable, accessible, real-time, and remote monitoring.

### µPAEDs for volatile organic compounds (VOCs)

Volatile organic compounds (VOCs) are generally defined as organic chemicals with a low boiling point and high vapor pressure at room temperature, ranging from simple hydrocarbons to alcohols and organic acids [113–115]. Although the basic definition of VOCs is unchanging, their bases of classification are in contention. Epping et al. state that VOCs can be categorized into biological and anthropogenic sources based on their origins. Biological or biogenic VOCs are often byproducts of metabolic and/or decomposition processes occurring in microorganisms, plants, and animals, whereas anthropogenic VOCs originate from incomplete combustions of fossil fuels, plastics, and aerosol products, perfumes, pesticides, tobacco smoke, etc. [113]. Others have categorized VOCs into exogenous and endogenous based on the source. In this case, endogenous VOCs are eliminated byproducts of metabolic reactions within the body, whereas exogenous VOCs are produced by external sources like microorganisms, plants, animals, food, and smoking [115]. Despite the confusion, it is largely agreed that most VOCs negatively impact human and environmental health, as they can be genotoxic, carcinogenic, mutagenic, and teratogenic. They may result in decreased crop yields, interference with natural rain precipitation and cloud formation, a high risk of damage to buildings and crops, global warming, and even impact human health in the long term [113]. VOCs are normally more concentrated in indoor environments than outdoor environments as they are emitted by carpets, furniture, paints, wallpapers, plastics like PVC, and via anthropogenic activities like cooking and smoking. Long-term and continuous exposure may severely damage human health [113, 116]. To prevent these problems and protect humans and the environment, quick and accurate detection of VOCs is necessary.

But before these compounds can be detected, they need to be collected from humans or the environment. Due to their nature, VOCs’ sampling offers an alternative to conventional sampling techniques. These volatile compounds are usually sampled from biological fluids like sweat, tears, saliva, urine, breastmilk, and, in some cases, skin and exhaled breath [117]. Through these biofluids, non-invasive sampling can be realized. This method is preferable compared to invasive sampling as it does not cause any distress to the patients and can be sampled and analyzed continuously and in real time. In this way, numerous samples can be collected in short time periods in a simplistic manner eliminating the need for help by medical staff and professionals [114, 118]. Moreover, collection of these samples is highly reproducible with no interference. In contrast, blood sampling is an invasive and unreliable technique due to evaporation of VOCs, which can lead to false measurement. In addition, accurate measurement of VOCs’ concentration within these samples is difficult due to the presence of interfering components [114, 118].

GC–MS and GCxGC-ToF are often considered to be gold standard methods for accurate detection of VOCs across several samples. They are sensitive and versatile making it possible to perform qualitative and quantitative assessments. In addition, these techniques have been exploited for their utility in identification of unknown VOCs [113, 118, 119]. Despite these advantages, they are unsuitable for use in remote and resource-limited areas due to their bulky equipment and non-transportability. Patients may have to bear additional costs as these techniques can only be carried out in laboratories. Additionally, they can only be tested on thermally stable VOCs with molecular weights less than 1000 Da [113, 118]. In light of this, researchers developed electrochemical sensors and electronic nose (EN) technology to overcome these issues. Electrochemical sensors operate by measuring the change in current produced by redox reactions between electrochemical cell and the analyte and thereby retrieve its qualitative and quantitative information [113]. On the other hand, ENs combine pattern recognition techniques with sensor arrays to analyze the unique olfactory fingerprints of various analytes. They have high sensitivity, specificity, and transportability and have been applied for continuous monitoring of conditions like lung cancer, diabetes, and renal diseases [113, 117, 118]. These techniques can be used in conjunction with nanomaterials like nanoparticles and nanorods, which enhance stability, response, and recovery times of the sensor [117]. Other point-of-care (POC) methods like paper-based microfluidics and µPAEDs have also been explored for their facile and rapid detection. These have been described below (Table 2).

**Table 2:** List of VOCs detected using µPAEDs along with extraction, printing, cutting*,* and detection methods

Sr. no	Analyte	Extraction method	Sample used	Type of paper used	Printing method	Cutting method	Detection method	LOD	Ref
1	Ethanol	Headspace extraction	Breath	Quantitative cellulose filter paper	Manual dip-coating	Manual cutting	Colorimetric	0.001 g/L	[120]
2	Hydrogen peroxide	Passive absorption	Simulated breath samples	Whatman No. 1 filter paper	Wax printing	Manual cutting	Electrochemical	Gaseous H_2_O_2_: 0.02 nA/µM/mm^2^	[121]
3	Benzaldehyde (BA)	Vapor generation paper-based thin-film microextraction	Exhaled breath samples from lung cancer patients and healthy volunteers	Cellulose-based paper	Vacuum filtration	Possibly manual cutting	Fluorescence and SERS	Fluorescence: 1.2 ppb SERS: 0.1 ppb	[122]
4	Ethanol	Headspace extraction	Animal blood samples (mice and sheep blood)	Whatman No. 4 filter paper	Screen printing with ink	Manual cutting or with precision machining tools	Digital image colorimetry	LOQ: 11.56 mg/dL	[123]
5	Cyanide	Headspace extaction	Blood samples from fire survivors, firefighters and healthy volunteers	Whatman Grade No. 2 filter paper	Laser printing with toner ink	Possibly manual cutting	Digital image colorimetry	0.4 µM	[124]
6	Formaldehyde (VOCs)	Headspace microextraction	Wastewater, textile, milk	Whatman Grade 44 cellulose filter paper	Laser printing	Laser cutting	Digital image colorimetry	0.03 mg/L	[125]
7	Ammonia (NH_3_) and sulfide (H_2_S)	Headspace extraction	Wastewater	For ammonia: Whatman No. 1 filter paper For sulfide: Whatman Grade 41 filter paper	NA	Manual cutting	Digital image colorimetry	Ammonia: 1.3 mg/L Sulfide: 1.7 mg/L	[126]
8	Ethanolamine (EA), dimethylamine (DMA) and trimethylamine (TMA)	Headspace extraction	River water	Whatman Grade 1 cellulose paper	Wax printing	Possibly manual cutting	Digital image colorimetry	EA: 0.4 ppm DMA: 0.2 ppm TMA: 0.5 ppm	[127]
9	Gaseous emissions of 3D filaments	Headspace extraction	3D filaments	Whatman No. 1 filter paper	Wax printing	NA	Colorimetric	NA	[128]

### Biological samples

Around 3000 VOCs across 874 types have been found in human breath including acetone, ethanol, isoprene, and ammonia [113, 115, 117, 118]. Most of these compounds originate through anthropogenic activities like fossil fuel emissions, and from plastics, traffic, and buildings. Other sources also include disinfectants, plasticizers, and volatile byproducts of drugs and other substances metabolized in the human body [119]. Following their production within the body, VOCs are transported to the lungs via systemic circulation and eliminated through exhalation [115]. The concentration of VOCs in exhaled breath is controlled by the body’s internal conditions, diet, lifestyle, and environmental exposure of humans to these sources. Jalal et al. refer to this concentration profile as “exposome.” By analyzing the olfactory profile of these exposomes, identification and prognosis of disease progression can be actualized. For example, acetonic smell and a pungent odor might be identified with hysterical diabetes and liver disease respectively [117]. Analyzing these olfactory fingerprints may be the key for revolutionizing breath analysis for disease diagnosis via development of POC-friendly biosensors and µPAEDs.

To materialize this, Mustafa et al. created a paper-based breath analyzer for instrument-free detection of ethanol in real exhaled breath samples. First, they immersed filter paper in a solution of cerium oxide nanoparticles (CeNPs) and cut it into discs after drying. Then, polyethylenimine (PEI) and alcohol oxidase (ALOx) were deposited in a sandwich style. Finally, the µPAED was placed into a hand-held device and exposed to the exhaled breath of volunteers. Ethanol vapors in the breath solubilize in PEI and interact with ALOx forming acetaldehyde and hydrogen peroxide. Hydrogen peroxide then reacts with CeNPs through a series of oxidative reactions yielding Ce-H_2_O_2_ complexes and a conversion from Ce^3+^ to Ce^4+^. The complexes generate a yellowish-brown color whose intensity was proportional to the concentration of peroxide, and thereby ethanol. Mustafa et al. also found that the µPAED performed similarly to, and sometimes even better than, commercial breath analyzers (LOD 0.001 g/L) [120]. Furthermore, the device showed no interference from other VOCs, with similar performance even after 120 days in optimum storage conditions. However, there are some issues which need to be resolved. Firstly, due to the usage of ALOx, the sensor’s specificity for ethanol is questionable as the reagent is non-specific. Secondly, the performance of the sensor may be affected if proper storage conditions are not maintained. Finally, the device has only been validated in a small population (*n* = 5), and its performance should be tested in a larger cohort.

**Fig. 4:**
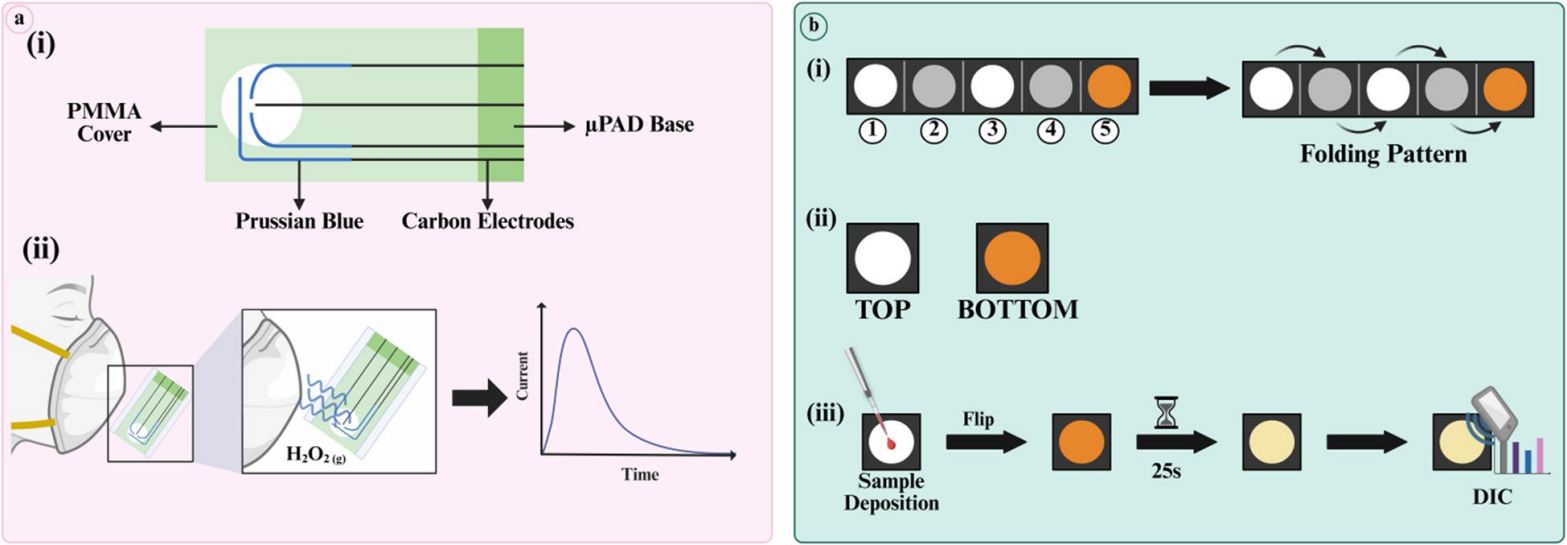
**a** (i) Depicts the final µPAED. Prussian blue was grafted onto carbon electrode surface. (ii) To complete the device, membrane strips were attached to its sides as shown in the figure. The completed µPAED was placed inside a respiratory mask to ensure even exposure to simulated breath samples. Hydrogen peroxide vapors are extracted from the breath via membrane strips. These react with Prussian blue–coated electrodes and oxidize Prussian blue, resulting in a change in amperometric properties (adapted from Ref. [121]). **b** (i) The overall design of the µPAED has been shown. The injection, acidification, gas collection, alkalization, and detection zones have been highlighted as (1), (2), (3), (4), and (5) respectively. TCA (white), buffer (gray), and receptor (orange) were deposited onto the zones. The µPAED was assembled by following the folding pattern as shown in the figure. (ii) Depicts the top and bottom layer of the completed µPAED. The top layer contains the sample injection zone, while the bottom layer contains the detection zone. (iii) After blood deposition, the µPAED is flipped. Then in the presence of cyanide, after 25 s, the orange color begins to fade. This image is captured and analyzed through digital image colorimetry (adapted from Ref. [124]) (created in https://biorender.com/)

Maier et al. designed a wearable paper-based breath analyzer for the detection of hydrogen peroxide in simulated breath samples (Fig. 4a). The paper was grafted with Prussian blue (PB)-modified carbon electrodes. The device was then placed inside a mask to ensure direct and even exposure to exhaled breath. Through passive absorption and diffusion, hydrogen peroxide vapors are extracted and absorbed onto the paper. These vapors interact with the electrodes and oxidize PB, generating an electrochemical signal. This signal was measured via amperometry. In this way, they applied a novel approach for calibration-free and sensitive detection of hydrogen peroxide (LOD 0.02 nA.µM/mm^2^) [121]. Additionally, this sensor is affordable, disposable, user-friendly, and wearable and allows for continuous and real-time analysis. For further improvement, this µPAED should be tested in real exhaled breath samples and in larger sample sizes.

Another µPAED has been built by Xia et al. for the detection of benzaldehyde (BA) in exhaled breath sampled from patients inflicted with lung cancer and from healthy volunteers. For this, they first prepared hybrids through self-assembly of gold nanorods (GNRs) and quantum dots (QDs). These were solubilized in methanol, stirred, ultrasonicated, and deposited onto the surface of µPAED through vacuum filtration. Then, BA was extracted from exhaled breath samples through nebulization and concentrated near its surface. These vapors diffused and permeated the paper, ultimately concentrating on the surface of GNRs-QDs hybrids through the cavity diffusion effect. This interaction interrupts the self-assembly of the hybrids and increases the fluorescence emissions of QDs. Thus, BA concentration and fluorescence emissions are directly correlated. Through this approach, a rapid dual detection system is realized, incorporating qualitative and quantitative assessment through fluorescence (FL) and SERS detection respectively (LOD: FL, 1.2 ppb; SERS, 0.1 ppb) [122]. However, this approach requires specialized equipment for analysis. Instead, DIC can be used. Although the µPAED is highly sensitive and selective, it still has a few issues that need to be addressed in future works. Firstly, this approach is limited to aldehyde-containing VOCs. Additionally, the usage of GNRs and QDs greatly increases the cost and complexity of fabrication, affecting accessibility, simplicity, and affordability of the device.

While exhaled breath analysis is gaining traction in disease diagnosis applications, analysis of VOCs in blood samples is also performed. Blood and plasma contain alcohols, alkanes, ethers, acetonitrile, ammonia, and benzyl hydrocarbons [117]. Most of these are produced by anthropogenic sources. In contrast, alcohols are produced via metabolic reactions under high stress conditions in the body. Through exposure to pollution, radiation, and smoking, high amounts of reactive oxygen species (ROS) are formed by mitochondria. In response, the body activates the detoxification process wherein cytochrome P450, a liver enzyme, oxidizes the compound through catalysis. The product formed is an alcohol, and an aldehyde is released as a byproduct [114]. Similar to exhaled breath analysis, VOCs can be detected in blood samples by analyzing their olfactory signatures. For example, elevated levels of chloroform may be associated with liver toxicity, anemia, and DNA damage, while an increase in benzene and toluene levels was observed in individuals prone to smoking [117].

Given their dynamic presence in blood, reliable and accurate detection methods are crucial for rapid, continuous, and real-time monitoring. This has led to significant innovation and advancements in the field. Thepchuay et al. designed and built a µPAED integrating headspace extraction and digital image colorimetry (DIC) for the detection of ethanol in animal blood samples. Using toner ink, they screen-printed the pattern onto filter paper and assembled the device which consisted of 3 layers. The front and back layers contained central reagent and sample reservoirs respectively. The middle layer was sandwiched between these two using double sided tape with a hole for the headspace. A mixture of ABTS, ALOx, and HRP was added to the reagent reservoir in the front layer. After drying, the µPAED was flipped and blood sample was loaded onto the sample reservoir in the back layer. To prevent ethanol leakage, both layers were covered with transparent tape. Ethanol vapors escape from the blood sample in the back layer to the headspace in the middle layer. The extracted ethanol then diffuses and permeates the reagent reservoir. Here, ALOx catalyzes its oxidation and produces acetaldehyde and hydrogen peroxide. The peroxide reacts with ABTS, a light green reagent, and is catalyzed by HRP producing a dark green color, which can be captured with a smartphone and analyzed using image analysis software. This µPAED was successfully used to detect ethanol in spiked animal blood samples with its performance being comparable to conventional HS-GC–MS, with high recovery (93.2% to 104.4%), high precision (RSD < 1%), rapid response time of 5 min, and large linear range (1–120 mg/dL) with low LOQ (11.56 mg/dL) [123]. Also, the device is portable, simple to use, affordable and offers good accuracy. However, the sensitivity of this device falls short when compared to conventional techniques. Also, it has only been tested in spiked samples. To get a view of its performance in the real world, it should also be tested against real and preferably human samples.

Sheini et al. also used a similar approach for designing their µPAED for the detection of cyanide in blood sampled from fire survivors and victims (Fig. 4b). Here as well, toner ink was used to laser print the pattern onto filter paper, which was grafted with a platinum (II) complex sensor. Then to extract cyanide from their blood, the samples were acidified with TCA. This breaks the bonds between cyanide and methemoglobin releasing HCN gas. These vapors are absorbed by borate buffer which deprotonates them, releasing free cyanide ions. These free ions interact with Pt(II) complex inducing a change in color from yellow to colorless. These were then analyzed by DIC. Through their novel approach, Sheini et al.’s µPAED was able to retrieve these results within 25 seconds with high sensitivity (LOD 0.4 µM) and selectivity [124]. They found that cyanide content was higher in fire victims than in fire survivors. Although the device is simple to use, it still involves a sample preparation step as plasma samples were used. To make it more user-friendly, the device may be modified to facilitate in-situ sample preparation on µPAED.

### Other samples

Other than biological samples, VOCs can and have also been collected from river water, wastewater, and food samples. Wastewater treatment plants generate high amounts of VOCs (at least 54 types) which can have drastic effects on human health upon long-term exposure including irritation, allergies, and an increased risk of cancer [129, 130]. VOCs can also permeate into various organs inside the body resulting in a myriad of diseases such as nervous, digestive, and hematopoietic diseases [131]. These compounds are released into the water by anthropogenic, industrial, and domestic activities through spills, urban run-offs, and improper disposal, affecting groundwater systems as well [129, 130, 132]. Some examples of VOCs found in wastewater samples include benzene, toluene, acetone, dichloromethane, carbon disulfide, formaldehyde, acetaldehyde, ethylbenzene, and xylene [129, 130]. To detect, identify, and measure their concentration in wastewater samples, novel detection techniques have been developed. Some notable applications have been mentioned below.

Mohammadi et al. integrated colorimetry and headspace microextraction (HSME) into a µPAD for instrument-free read out of formaldehyde in wastewater, milk, and different textile samples. The final µPAD consisted of two layers. Both these layers were fixed onto PMMA backing sheets to prevent reagent leakage. The bottom layer had sample and donor zones, while the top layer consisted only of a detection zone superimposed onto the donor zone. This detection zone was pre-deposited with a derivatization reagent. After deposition and attachment of the two layers, the µPAED was placed inside a dark box and heated. By heating the device, VOCs vaporize from the sample and adsorb onto the fibers on the detection zone. Here, the VOCs react with the derivatization reagent through the Hantzsch reaction forming a yellow-colored product after 3 min. Images were captured with smartphone and analyzed with image analysis software. This device offers rapid testing and is portable, accessible, affordable, sensitive (LOD 0.03 mg/L) and selective [125].

**Fig. 5:**
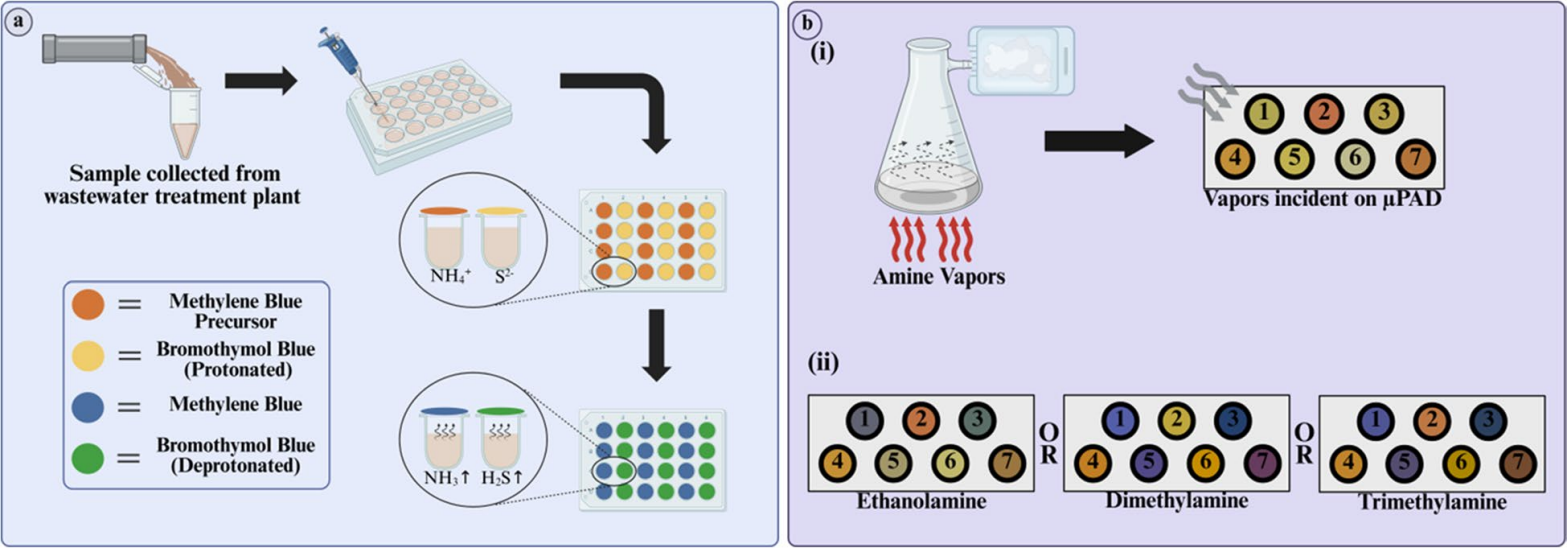
**a** Samples were collected from wastewater treatment plants and pipetted into microwell plate. µPAEDs were fabricated by manual dipping of filter paper into methylene blue precursors (orange) and bromothymol blue (yellow) for the detection of ammonia and sulfide respectively. After loading of NaOH and HCl solutions, the microplate was sealed with µPAEDs and PTFE membrane (not depicted here). After vaporization of the analytes, a color change is prompted by their interaction with the reagents. A color change from orange to blue was observed for ammonia, whereas a color change from yellow to green was observed for sulfide (adapted from Ref. [126]). **b** (i) Amine vapors are generated by heating amine liquid. These vapors are collected in a gas sampling bag and incident on the µPAED in a sensing chamber (not depicted). The final µPAED contained 7 rings which were pre-deposited with various dyes. These are bromophenol blue (1), methyl red (2), bromocresol green (3), disperse orange 3 (4), bromocresol purple (5), fluorescein (6), and cresol red (7). (ii) Olfactory fingerprints of the various amine vapors have been depicted with their respective changes in colors (adapted from Ref. [127]) (created in https://biorender.com/)

Vargas-Muñoz et al. created and designed a µPAED for dual detection of ammonia (NH_3_) and sulfide (H_2_S) vapors in wastewater samples (Fig. 5a). Instead of using wax or toner ink for printing, the filter paper was soaked in the detection reagents specific to the analytes. Bromothymol blue (BTB) and methylene blue precursor reagents were used for ammonia and sulfide detection, respectively. These were cut into discs using a paper puncher. As seen in previous cases, HSE was performed for VOCs extraction. The vapors then diffused through a membrane onto the discs. Ammonia vapors react with BTB causing a color change from yellow to green, whereas aqueous sulfide in the sample reacts with methylene blue precursors forming methylene blue. The sensor exhibited results comparable to conventional detection techniques and showed good precision with high recovery rates. In addition, it is sensitive (LOD: NH_3_, 1.3 mg/L; H_2_S, 1.7 mg/L), eco-friendly, affordable, and portable and is capable of high-throughput performance [126]. However, it is possible that the device may not detect analyte concentrations less than the LODs. Also, color generation on the discs may be affected by changes in humidity and moisture.

VOCs have also been found in aquatic ecosystems. They generally originate from biological organisms like algae and cyanobacteria, from anthropogenic activities like construction, and from industrial, domestic, urban, and agricultural sources. Other sources include run-offs from storm drains and wastewater treatment facilities and fuel and exhaust emissions from maritime transport facilities [133]. Benzene, toluene, ethylbenzene, and xylene (BTEX) are some of the most commonly emitted VOCs from these sources. These compounds are easily absorbed, distributed, and accumulated in well-vascularized organs and systems like respiratory, cardiovascular, urinary, digestive, and reproductive. Long-term exposure to BTEX can result in complications in the central nervous system, immune system, and reproductive systems and is often associated with respiratory tract infections, chest inflammation, reduced lung function, asthma, and increased risks of lung cancer [134, 135]. Some examples of commonly found VOCs in river water samples include ethanolamine, BTEX, acrylamide, ketones, and esters [133, 134].

Recently, Nguyen et al. designed a device for detecting amine-containing VOCs (ethanolamine (EA), dimethylamine (DMA), and trimethylamine (TMA)) in river water samples (Fig. 5b). A pattern with 7 rings was printed onto filter paper via wax printing. Then, the rings were pre-deposited with a variety of pH indicators and cut into sheets. These sheets were stored inside a sensing chamber. To generate amine vapors, samples were heated in a flask and vapors were collected in a sampling bag. These were transferred to the sensing chamber. When these vapors interact with detection zones in µPAED, a change in pH was observed which engendered a color change. This µPAED could effectively distinguish between 3 classes of amine-VOCs in real samples with high recovery rates (94.8% to 108.7%) and no interference from common VOCs. In a nutshell, the device is affordable, rapid, portable, accessible, disposable, and sensitive (LOD: EA, 0.4 ppm; DMA, 0.2 ppm; TMA, 0.5 ppm) and requires minimal sample preparation [127], but its performance may be affected by changes in humidity.

As mentioned previously, most VOCs, including alcohols, aldehydes, siloxanes, and aromatic hydrocarbons, are detected in indoor environments which is mainly due to emission source [136]. Building materials, furniture, cleaning agents, personal care products, perfumes, aerosols, exhaled breath, and skin emissions are some sources of VOC emissions [136, 137]. Additionally, variations in environmental temperature and humidity, human and household activity, and population density have also been proven to contribute to higher emissions in such environments [136, 137]. Through long-term and continuous exposure to VOCs, humans face higher risks of damaging their health [136]. Some effects include skin irritation, headaches, difficulty breathing, asthma, wheezing and an amplified vulnerability to cancer [136, 137].

In a recent study, Pinheiro et al. developed a paper-integrated optical µPAED for the detection of gaseous emissions of 3D printing filaments. The pattern consists of 15 detection zones which were pre-deposited with a variety of dyes. Then, using a 3D-printer and an array of 3D filaments, several items were printed. To ensure uniform and even exposure to VOCs, the sensor was placed inside the printer. When the filaments were heated during printing, VOCs adsorbed onto the detection zones. They interacted with the various dyes in these zones, resulting in color changes which were analyzed using image analysis software. Pinheiro et al.’s novel instrument identified that acrylonitrile–butadiene–styrene (ABS) filaments emitted several VOCs and increased the risk of indoor air pollution. Overall, this sensor is disposable, affordable, portable, versatile, reproducible, and rapid and shows potential for DIC [128]. However, in this work, it has only been used for qualitative detection. It therefore remains uncertain whether it can be used for quantitative measurements. This uncertainty is only exacerbated due to its non-specificity. Most of the dyes used for detection can detect aldehydes, ketones, amines, or carboxylic acids—which are common across several VOCs. In addition, Pinheiro et al. have only tested the efficiency of this sensor in a singular printer. Since different printers have differing settings, the device may not perform similarly and may need to be calibrated prior to use.

### Conclusion and future perspectives

Biomarker and chemical contaminant monitoring are important applications in disease diagnostics. By studying their concentrations within the body, scientists can glean information about the well-being and health of the patient. Alternatively, it has been used for the identification and prognosis of various diseases. Thus, rapid and timely detection is necessary to prevent fatality and protect human health and welfare. For this reason, several conventional techniques were developed. However, most of these techniques involved usage of bulky equipment, experienced handling, and were exceedingly expensive despite their high precision, accuracy, and sensitivity. To overcome this, point-of-care techniques like microfluidics and lateral flow immunoassays were developed. In this review, microfluidics and µPADs were considered.

To detect analytes in various samples, sample preparation steps are needed. Thus, the most common approach utilized conventional extraction techniques like solid-phase extraction, in conjunction with µPADs. Extracts of samples were prepared through these techniques and analyzed for biomarker/chemical contaminants using µPADs. But several conventional extraction techniques require complex equipment, experienced handling, and are usually performed in laboratories. Although µPADs may be simple and user-friendly, through this approach, sample pretreatment must be done separately. Thus, the multitudinous benefits of µPADs are overshadowed by the need for centralized laboratories. Overall, this approach of combining µPADs and conventional extraction techniques is not feasible for on-field testing.

As a result, several researchers took up the mantle of innovating an integrated all-in-one device that is truly decentralized in its application. This led to the creation of microfluidic paper–based analytical extraction devices (µPAEDs). These devices are portable and affordable and offer on-site extraction and detection. As compared to the usual approach, it is faster and more efficient. In addition, µPAEDs demonstrate a potential for equitable access and commercialization due to their simple and miniature design features. However, µPAEDs only follow part of the “ASSURED” criteria (affordable, sensitive, specific, user-friendly, rapid and robust, equipment-free, and deliverable to end-users) as set by the WHO [138]. They must be further developed to be truly equipment-free and user-friendly. The latest developments in this area were also discussed for both biomarker and chemical contaminant monitoring. However, for these applications, most of the biological samples were collected through invasive techniques. Such samples include blood, serum, interstitial fluid, and throat swabs [139]. These methods cause distress to patients and require knowledge of medical professionals for sampling. Additionally, samples cannot be collected continuously. Therefore, researchers shifted their focus to non-invasive sampling methods and analytes which could be detected in such samples. For this review, VOCs were discussed.

Volatile organic compounds or VOCs have seen a recent rise in popularity due to their ease of collection through non-invasive means in biological samples such as breath, tears, and sweat. In this approach, samples can be collected continuously without causing harm or distress to the patient. In fact, breath samples can be collected from unconscious patients as well. Moreover, real-time and continuous monitoring is made possible. This is a significant step forward in the future of disease diagnostics. In this review, recent advances in VOC detection across a myriad of samples using µPAEDs were also highlighted.

But, this is only the tip of the iceberg. Through further investigation and innovation, this approach can be designed for wearable device applications. Additionally, it can be integrated with artificial intelligence and machine learning techniques, forming a smart wearable device. This will make µPAEDs more accessible to the general population. Furthermore, conventional methods can also be miniaturized and used together with µPADs and/or µPAEDs to enhance their portability and overall accuracy, precision, and sensitivity.

## Data Availability

No datasets were generated or analysed during the current study.

## Abbreviations

µPAD: Microfluidic paper–based analytical device
µPAED: Microfluidic paper–based analytical extraction device
2,4-D: 2,4-Dichlorophenoxyacetic acid
3-NT: 3-Nitrotyrosine
4-AAP: 4-Aminoantipyrine
4-NQO: 4-Nitroquinoline-N-oxide
AA: Ascorbic acid
ABS: Acrylonitrile-butadiene-styrene
ABTS: 2,2′-Azino-bis(3-ethylbenzothiazoline-6-sulfonic acid)
AChE: Acetylcholinesterase
ACN: Acetonitrile
ALOx: Alcohol oxidase
APTES: (3-Aminopropyl)triethoxysilane
AuNPs: Gold nanoparticles
BA: Benzaldehyde
BEST: Biomarkers, endpoints, and other tools
BTB: Bromothymol blue
BTEX: Benzene, toluene, ethylbenzene, and xylene
CdTe-QD: Cadmium telluride quantum dots
CEA: Carcinoembryonic antigen
CeNPs: Cerium oxide nanoparticles
CMIP: Conductive molecularly imprinted polymer
COPD: Chronic obstructive pulmonary disease
CQD: Carbon quantum dots
CV: Cyclic voltammetry
d-SPE: Dispersive solid phase extraction
DDTC: Sodium diethyldithiocarbamate
DI: Deionized water
DIC: Digital image colorimetry
DMA: Dimethylamine
DNA: Deoxyribonucleic acid
DPV: Differential phase voltammetry
d-SPE: Dispersive solid phase extraction
EA: Ethanolamine
ECF: Extracellular fluid
EIS: Electrochemical impedance spectroscopy
ELISA: Enzyme-linked immunosorbent assay
EME: Electromembrane extraction
EN: Electric nose
FDA: U.S. Food and Drug Administration
FL: Fluorescence
GC–MS/MS: Gas chromatography-tandem mass spectrometry
GC-MS: Gas chromatography-mass spectrometry
GCxGC ToF: Comprehensive two-dimensional gas chromatography-time-of-flight mass spectrometry
GNRs: Gold nanorods
GQDs: Graphene quantum dots
HMN: Hollow microneedle extraction
HPLC-DAD: High-performance liquid chromatography with diode array detection
HRP: Horseradish peroxidase
HSE: Headspace extraction
HS-GC-MS: Headspace gas chromatography-mass spectrometry
HSME: Headspace microextraction
ICP-MS: Inductively coupled plasma mass spectrometry
IIP: Ion-imprinted polymer
IPA: Isopropyl alcohol
ISF: Interstitial fluid
LAMP: Loop-mediated isothermal amplification
PDMS: Polydimethylsiloxane
PMMA: Polymethylmethacrylate
PTSD: Post-traumatic stress disorder
qPCR: Quantitative polymerase chain reaction
QuEChERS: Quick, easy, cheap, effective, rugged and safe
TBA: Thiobarbituric acid
WHO: World Health Organization
